# Guidelines for Rapport-Building in Telehealth Videoconferencing: Interprofessional e-Delphi Study

**DOI:** 10.2196/76260

**Published:** 2025-08-07

**Authors:** Paula D Koppel, Jennie C De Gagne, Michelle Webb, Denise M Nepveux, Janelle Bludorn, Aviva Emmons, Paige S Randall, Neil S Prose

**Affiliations:** 1 School of Nursing, Duke University, DUMC 3322, 307 Trent Drive, Durham, NC, 27710, United States, 1 617 835 7087; 2 School of Medicine, Duke University, Durham, NC, United States; 3 Duke Center for Interprofessional Education and Care, Duke University, Durham, NC, United States; 4 Duke Cancer Institute, Durham, NC, United States; 5 Pediatrics and Dermatology, Global Health Institute, Duke University, Durham, NC, United States

**Keywords:** health professions education, health care professional development, clinician-patient relationship, interpersonal skills, health care communication, empathy, web-based care

## Abstract

**Background:**

Telehealth training is increasingly incorporated into educational programs for health professions students and practicing clinicians. However, existing competencies and standards primarily address videoconferencing visit logistics, diagnostic modifications, and etiquette, often lacking comprehensive guidance on adapting interpersonal skills to convey empathy, cultural humility, and trust in web-based settings.

**Objective:**

This study aimed to establish consensus on the knowledge, skills, and attitudes required for health professions students and clinicians to build rapport with patients in telehealth videoconferencing visits and to identify teaching strategies that best support these educational goals.

**Methods:**

An e-Delphi study was conducted using a panel of 12 interprofessional experts in telehealth and telehealth education. Round 1 involved interviews, followed by anonymous surveys in rounds 2‐4 to build consensus.

**Results:**

All 12 experts participated in rounds 1‐3. In total, 19 themes related to rapport-building and 77 specific curriculum items were identified, all achieving the established level of consensus.

**Conclusions:**

Using a competency-based education framework, this study provides guidance for health professions educators, teaching clinicians, and students on how to adapt interpersonal skills for telehealth including detailed content related to knowledge, skills, attitudes, and teaching strategies. Future research is needed to test the feasibility, acceptability, and effectiveness of curricula based on these competencies and teaching strategies.

## Introduction

### Background

Telehealth, including the use of videoconferencing visits (VV), is quickly becoming an important and common vehicle for delivering health care [1,2]. Growth in the use of VV is anticipated, given that clients or patients (hereafter referred to collectively as patients) and clinicians are increasingly receptive to VV as a supplement to in-person care [2].

Although telehealth competencies and training are being incorporated into some health professions educational programs [3-7], a recent scoping review found that 53% of sources emphasized a need for additional education and practice in telehealth etiquette and interpersonal aspects of care [8]. Many health care professionals remain uncertain of ways to establish rapport during VV [9-11]. A mixed methods study by Elsevier Health that included 3000 nurses and doctors found that over half believed telehealth would negatively impact their ability to demonstrate empathy and requested further guidance on interpersonal skills in VV [12]. Studies show that not all interpersonal skills are interchangeable between in-person and VV [2,13] and that adapting skills for VV often does not come naturally but can be learned [14,15].

### Definition of Rapport and Importance

Rapport, a term often used colloquially, has been defined within the health sciences and psychology as a desirable state of interpersonal connection that requires positivity, mutual responsiveness, and behavioral coordination [16,17] as well as respect, acceptance, empathy, and a mutual commitment to engage [18]. When intentionally fostered, rapport facilitates the development of a therapeutic relationship with outcomes that include trust, improved care outcomes, and satisfaction for both the patient and clinician [10, 13, 19-23]. Connection or rapport within a web-based care environment has only begun to be explored. These studies suggest that many in-person relationship-based skills are effective, but adaptations are necessary and must be used intentionally by clinicians and patients [9,10,20,24].

### Knowledge Gap and Need for Research

Recent evidence-based telehealth etiquette and interpersonal checklists [25,26], along with educational interventions for health care students [14] and professional development workshops for clinicians [15,27], show promise in preparing current and future clinicians to adapt their interpersonal skills. However, telehealth etiquette often emphasizes practical and professional behaviors for efficient VV (eg, technical preparedness, ensuring a professional environment, and communication techniques to ensure diagnostic and treatment accuracy) rather than adapting interpersonal skills to convey empathy, cultural humility, and trust or to build personal connection in a web-based environment. Additionally, evidence-based resources are needed for interprofessional educators to support curriculum development across health care disciplines [7].

Building rapport in VV requires specific knowledge, skills, and attitudes as well as teaching strategies designed to facilitate relationship-based care competencies. Building upon previous descriptive studies [9,10,13,20,28-31], this project aimed to use an e-Delphi study to explore what relationship-based care elements are important to include in a telehealth curriculum. This will provide a strong foundation for the future curricula development of interprofessional students and clinicians across disciplines while closing a crucial gap in our practice and pedagogical knowledge.

### Research Aim and Questions

This study aimed to establish expert consensus on training health professions students and clinicians in rapport-building during VV. Two research questions guided the study: (1) what knowledge, skills, and attitudes are essential for health professions students and clinicians to build rapport with patients in telehealth VV? (2) What teaching strategies best facilitate the development of this knowledge and these skills and attitudes?

## Methods

### Study Design

An e-Delphi methodology was used to build consensus by collecting, analyzing, and sharing data results with an interprofessional panel of experts in telehealth and telehealth education. The Delphi technique, widely used in nursing and health care research, assumes that group opinion is more valid and reliable than individual opinion, making it effective for achieving consensus on topics with limited evidence or uncertain practice standards [32]. This iterative, structured method uses interviews and surveys to gather expert input over multiple rounds until consensus is reached. Experts remain anonymous to one another to prevent any individual from dominating the process. After each survey round, data are summarized and shared with experts, reflecting both their individual responses and the group’s collective responses. This feedback allows experts to revise their input based on group insights. Rounds typically continue, often between 2 and 4 times, until an acceptable level of consensus is achieved [32]. The web-based Delphi technique (e-Delphi), increasingly adopted for its cost-effectiveness, facilitates efficient participation from geographically diverse experts [33].

### Recruitment and Selection of Participants

An interprofessional panel of national and international experts was recruited to participate in this e-Delphi study. The panel included licensed nursing and medical professionals (registered nurses, medical doctors, and advanced practice providers), as well as speech and occupational therapists, all proficient in reading, writing, and conversing in English. Participants were required to have experience in at least 1 of the following activities: (1) conducted research on VV, (2) participated in telehealth advisory panels, (3) developed telehealth curricula, or (4) provided extensive instruction in telehealth, with a significant focus on adapting relational and communication skills for telehealth care.

Our interprofessional research team initially identified 21 experts meeting these criteria. These experts were invited to recommend additional participants, resulting in 7 further suggestions. Recruitment occurred over 3 rounds of targeted emails to assemble a diverse panel of telehealth experts with broad perspectives and experiences. To encourage participation across all rounds of the study, participants received a small financial incentive for their time upon completion.

### Ethical Considerations

This educational research was deemed to pose minimal risk to the expert participants, as defined under the Common Rule; therefore, written consent was not conducted. A statement in the initial questionnaire clarified that participants’ willingness to complete the interview and surveys constituted their verbal consent. The study received an exempt determination from the health system’s institutional review board for clinical investigations (protocol ID: Pro00117125) prior to beginning the study. To maintain participant privacy, data were deidentified, and all study communications, interviews, and study data were conducted and stored on a secure password-protected server with firewall protection and multifactor authentication. Additionally, the study was registered in the Open Science Framework on January 22, 2025 [34].

### Data Collection Procedures

Four rounds of data collection were planned to build adequate consensus among the experts. In round 1, interviews were conducted with experts individually on a university-approved secure Microsoft Teams videoconferencing platform using an interview guide with semistructured open-ended questions developed by the research team. This guide, informed by the 2 original research questions, focused on the knowledge, skills, attitudes, and teaching strategies essential for building rapport in VV. Interviews were chosen as a method for the initial round to gather qualitative descriptive data based on the experts’ experiences, given the limited published research on rapport-building in VV.

The specific knowledge, skills, attitudes, and teaching strategies identified using qualitative content analysis of the interview data in round 1 were compiled into a Qualtrics survey for round 2. The round 2 survey asked experts to prioritize the knowledge, skills, attitudes, and teaching strategies using a 4-point Likert scale by asking the extent of importance (4=very important, 3=important, 2=of some importance, and 1=not important). A 4-point scale was selected to enhance survey completion and encourage participants to choose between an item being important or unimportant [35]. Contextually, this aligns with an educator’s need to decide whether to include or exclude content from their curriculum. Open-ended questions allowed the participants to provide comments related to their decisions and describe any need to revise or expand the content.

The round 3 survey included the same questions as round 2 and was designed to show experts their ratings compared to those of the other experts in the study. The experts were given an opportunity to rerate each of their original responses or keep them the same. Revision of round 2 items or recommendations for new items from open-ended questions in round 2 were also incorporated into the round 3 survey. Member checking using a final questionnaire was planned in round 4 to evaluate if the panel felt the results reflected their expertise and recommendations. All Qualtrics surveys were sent to participants via the institution’s secure Microsoft Teams platform.

Expert participants received emails with survey links for all rounds of the study, including a questionnaire to collect their relevant professional background details prior to the first-round interview. Surveys were developed and administered using Qualtrics, a secure university-approved web-based survey platform.

### Data Analysis and Statistical Considerations

Descriptive statistics were selected to report panel responses for each survey item (mean, SD, IQR, and consensus level) and attrition rates over the course of rounds. Since building a curriculum necessitates making a dichotomous decision to include or exclude topics or teaching strategies, for the purposes of calculating consensus, responses were collapsed into 2 categories: important (items rated very important or important) and unimportant (items rated less important or unimportant). The percentage of agreement between the experts was used to measure the level of consensus. Although curricula to teach rapport-building in VV may not require complete agreement, rapport is an important indicator of quality care; therefore, consensus was considered sufficient when an item was rated important or very important by at least 70% of the experts on the panel [32,36]. Statistical analysis was supported by using Microsoft Copilot within Microsoft Excel, leveraging its artificial intelligence (AI)–driven capabilities to generate insights and summaries.

A 5-member coding team conducted qualitative content analysis of the narrative interview data from round 1 and the open-ended responses for all later survey rounds. Codes were derived directly from the transcribed text data and kept close to the participants’ descriptions [37], and the analysis was guided by a process outlined by Elo and Kyngäs [38]. NVivo software (version 14.0; QSR International Pty Ltd) was used to facilitate the qualitative analysis, including coding and development of a codebook. At least 20% of the data were reviewed by 2 or more team members, with conflicts resolved through discussion. After initial manual coding, the team used Copilot to generate preliminary summaries and thematic overviews of the interview data. Testing and refining prompts ensured that results aligned with the manually generated NVivo codebook. The study’s rigor was enhanced by (1) team-based analysis by researchers with extensive qualitative experience; (2) judicious use of AI tools to validate findings [39]; (3) regular coding meetings to refine codes, categories, themes, and survey items [40]; (4) concurrent data collection and analysis [41]; (5) detailed memos to maintain an audit trail of analytical decisions [39]; and (6) member checking by asking participants to clarify interview responses, evaluate survey items, and reflect on the final analysis [42]. The ACCORD (Accurate Consensus Reporting Document) guided the reporting of results [43].

## Results

### Overview

In total, 12 of the 16 interprofessional telehealth experts emailed an invitation consented to participate in the study, representing a 75% response rate. Interviews were conducted with the panel of experts in January 2025. This was followed by 3 survey rounds from February to April 2025. All 12 experts completed the round 1 interview and the round 2 and round 3 surveys, representing a 100% participation rate. A total of 11 of the 12 (92%) experts participated in round 4 member checking.

### Panel Characteristics and Expertise

Most participants had doctoral degrees (8/12, 67%) and more than 20 years of professional experience (8/12, 67%). A third of the experts had 11 or more years of experience using telehealth with a deep range of involvement in practice, education, research, and advisory capacities (see Table 1 for further characteristics). Of the 12 experts, 2 (17%) were international experts, with the balance residing in the United States. The panel’s expertise included using telehealth in a variety of contexts, including adult and pediatric care, rural settings, palliative and hospice care, and mental health. Members of the expert panel have contributed to the development of practice standards and educational curricula, established telehealth clinics, taught educational courses, conducted research, and published journal papers and books on telehealth. In addition, several experts in the study have developed conceptual models and conducted research specifically on relational aspects of telehealth. All participants were actively involved in teaching professional and interprofessional educational programs with a focus on telehealth.

**Table 1.** Expert participant characteristics (N=12).

Characteristic		Values, n (%)
Education level		
	Master’s degree	2 (17)
	Doctoral degree	8 (67)
	Professional degree (MD)	2 (17)
Licensure		
	Nursing or midwifery	7 (58)
	Medicine	2 (17)
	Speech-language pathology	1 (8)
	Physician assistant	2 (17)
Length of professional experience (years)		
	11‐12	4 (33)
	21‐30	3 (25)
	31‐40	4 (33)
	>40	1 (8)
Professional experiences with telehealth		
	Conducted visits or consultations	9 (75)
	Conducted research	10 (83)
	Participated on advisory panels	8 (67)
	Developed curriculum	8 (67)
	Provided instruction focused on rapport	11 (92)
Length of telehealth experience (years)		
	2‐4	4 (33)
	5‐10	4 (33)
	11‐20	4 (33)

### First Round Results

#### Overview

Data collected and coded from the 12 expert interviews were organized into educational areas of knowledge, skills, attitudes, and teaching strategies as directed by the research question. Within each of these areas, themes emerged that helped organize more specific items that the experts recommended for a curriculum focused on building rapport in VV. The themes and specific items related to each topic are described below. Since these educational areas, themes, and specific items were incorporated into the round 2 survey, they can also be reviewed in their entirety in Tables 2-5, along with their descriptive statistics and consensus ratings.

**Table 2.** Education areas within the knowledge domain (N=12).

Theme and item		Consensus^a^, n (%)	Mean (SD)^b^	Median (IQR)
Basic knowledge of telehealth and its applications				
	Telehealth taxonomy	9 (75)	2.83 (0.835)	3 (2.25-3)
	Evidence-based uses of video visits	12 (100)	3.83 (0.389)	4 (4-4)
	Types of video visits (eg, urgent care, follow-up care, and medical management)	12 (100)	3.50 (0.522)	3.5 (3-4)
	Types of clinician roles and responsibilities (eg, clinical evaluation, preoperative education, and facilitating support group)	11 (92)	3.33 (0.651)	3 (3-4)
	Situations not appropriate for video visits	12 (100)	3.75 (0.452)	4 (3.25-4)
	Common challenges in video visits	12 (100)	3.92 (0.289)	4 (4-4)
	General adaptations necessary	12 (100)	3.58 (0.515)	4 (3-4)
How to create a web-based environment that supports rapport				
	Ground rules for video visits (eg, what to expect, what to do if internet connection lost, and ways to enhance the quality of the visit)	12 (100)	3.83 (0.389)	4 (4-4)
	Awareness of how generational, cultural, educational, geographic, and socioeconomic factors influence a patient’s participation in video visits	12 (100)	3.75 (0.452)	4 (3.25-4)
	Importance of privacy (auditory or visual) and confidentiality in the patient’s and clinician’s spaces (eg, private spaces and data protection)	12 (100)	3.92 (0.289)	4 (4-4)
	Choice of video visit setting or background (eg, quiet and nondistracting)	12 (100)	3.50 (0.522)	3.5 (3-4)
	Adaptations related to patient population or type of visit (eg, children, non-English speakers, team-based consultation, mental health evaluation, and group-based care)	12 (100)	3.75 (0.452)	4 (3.25-4)
Functional use of videoconferencing technology platforms				
	Understanding of videoconferencing platform functions and set-up	12 (100)	3.33 (0.492)	3 (3-4)
	Problem-solving of most common technical problems	12 (100)	3.25 (0.452)	3 (3-3.75)
Rapport basics				
	Awareness of attributes of rapport (eg, shared experience of comfort and engagement and being “in sync”)	12 (100)	3.83 (0.389)	4 (4-4)
	Awareness of the outcomes of rapport for patients and clinicians (eg, trust and collaboration)	12 (100)	3.83 (0.389)	4 (4-4)
	Basic rapport-building strategies (eg, active listening and showing genuine interest, respect, and empathy)	12 (100)	4.00 (0.000)	4 (4-4)
	Importance of patients feeling heard and understood	12 (100)	4.00 (0.000)	4 (4-4)
	Increased importance of facial expression and body mannerisms in video visits	12 (100)	3.67 (0.492)	4 (3-4)
	Indicators that rapport has been established (eg, shared smiles, laughter, warmth, and sense of connection)	12 (100)	3.83 (0.389)	4 (4-4)
	Unique opportunities to cultivate rapport in video visits	12 (100)	3.67 (0.492)	4 (3-4)
	Unique rapport challenges in video visits (eg, internet lag interfering with the ability to feel “in sync” and visits becoming brief and transactional)	12 (100)	3.83 (0.389)	4 (4-4)

a This describes the percentage of experts who scored the item as either “important” or “very important.”

b Group mean Likert score. Note that each specific item within the survey was rated as 4=very important, 3=important, 2=of less importance, or 1=unimportant.

**Table 3.** Education areas within the skills domain (N=12).

Theme and item		Consensus^a^, n (%)	Mean (SD)^b^	Median (IQR)
Creating a safe and comfortable web-based environment				
	Supporting patient’s comfort with technology	11 (92)	3.58 (0.669)	4 (3-4)
	Managing technical challenges	11 (92)	3.33 (0.651)	3 (3-4)
	Ensuring patient’s desired level of privacy during the video visit	12 (100)	3.83 (0.389)	4 (4-4)
	Navigating technological and other video visit distractions to maintain rapport	12 (100)	3.67 (0.482)	4 (3-4)
	Taking time to build rapport at the beginning of the video visit	12 (100)	3.92 (0.289)	4 (4-4)
	Monitoring level of rapport throughout the video visit	12 (100)	3.50 (0.522)	3.5 (3-4)
	Navigating the patient’s wants and needs within video visit parameters with mutually agreeable care goals	12 (100)	3.83 (0.389)	4 (4-4)
	Demonstrate knowing the patient with recall of important information (eg, medical and social)	12 (100)	3.58 (0.515)	4 (3-4)
	Demonstrating cultural humility (eg, respecting differences and practicing self-awareness)	12 (100)	3.67 (0.492)	4 (3-4)
	Managing time and flow so patients do not feel rushed	12 (100)	3.50 (0.522)	3.5 (3-4)
	Avoiding abruptly ending visits, ensuring that patients' questions and needs are addressed	12 (100)	3.92 (0.289)	4 (4-4)
	Navigating moments of disconnection (eg, anger, disagreement, and disengagement)	12 (100)	3.75 (0.452)	4 (3.25-4)
Adapting verbal communication techniques to facilitate rapport in video visits				
	Keeping communication clear and simple	12 (100)	3.67 (0.492)	4 (3-4)
	Managing conversation flow and pacing to reduce interruptions and “talk overs”	12 (100)	3.75 (0.452)	4 (3.25-4)
	Using words as a replacement for physical touch to acknowledge emotions and demonstrate support (eg, “I wish I could give you a hug”)	11 (92)	3.58 (0.669)	4 (3-4)
	Increasing use of reflective practices (eg, restating what the patient has said, asking more questions, and using teach-backs) to validate or confirm accurate understanding	12 (100)	3.83 (0.389)	4 (4-4)
	“Narrating the visit” to describe what you are doing that might be difficult for the patient to interpret (eg, looking at your laboratory work and writing orders for medication)	12 (100)	3.75 (0.452)	4 (3.25-4)
Enhancing nonverbal communication techniques to facilitate rapport in video visits				
	Using body language (eg, facial expressions, hand gestures, and body posturing) to express feelings, emphasize suggestions, or show intentions (eg, eye contact, nodding, and leaning in to show attentiveness, smiling, and hand over heart to show empathy)	12 (100)	4.00 (0.000)	4 (4-4)
	Using platform technology to keep the patient engaged and enhance learning or understanding (eg, screen sharing images, placing links to information in chat, and “show and then tell”)	11 (92)	3.50 (0.674)	4 (3-4)
Maintaining attentiveness and presence				
	Pausing or breaking between video visits to reset and cultivate presence	10 (83)	3.17 (0.718)	3 (3-4)
	Managing personal distractions (eg, phone and computer alerts)	12 (100)	3.58 (0.515)	4 (3-4)
	Setting up technology and lighting to ensure that both the clinician and the patient can view the other’s body language (eg, “passport view”)	12 (100)	3.58 (0.515)	4 (3-4)
	Setting up technology to enhance eye contact with the patient during documentation and medical record review (eg, close to the computer camera)	12 (100)	3.75 (0.452)	4 (3.25-4)
	Heightening attentiveness to visual and auditory signals that might reflect patient emotion	12 (100)	3.58 (0.516)	4 (3-4)
Using a web-based environment to humanize the care experience				
	Seeking cues in the patient’s environment to know them as a person	10 (83)	3.25 (0.754)	3 (3-4)
	Using cues in the patient’s environment to build connection	10 (83)	3.25 (0.754)	3 (3-4)
	Using self-disclosure based on cues in the patient’s environment to build connection	9 (75)	2.92 (0.900)	3 (2.25-3.75)
	Personalizing your professional web-based environment to facilitate connection	9 (75)	3.00 (0.739)	3 (2.25-3.75)
	Using idiosyncratic aspects of video visits as opportunities to build connection (eg, unexpected pet encounters or technology issues)	12 (100)	3.25 (0.452)	3 (3-3.75)
Other				
	Magnifying use of active listening techniques	12 (100)	3.83 (0.389)	4 (4-4)
	Heightening self-awareness to ensure that your verbal and nonverbal behaviors reflect empathy and caring	12 (100)	3.92 (0.289)	4 (4-4)

a This describes the percentage of experts who scored the item as either “important” or “very important.”

b Group mean Likert score. Note that each specific item within the survey was rated as 4=very important, 3=important, 2=of less importance, or 1=unimportant.

**Table 4.** Education areas within the attitudes domain (N=12).

Theme and item		Consensus n (%)^a^	Mean (SD)^b^	Median (IQR)
Willingness to adapt to changing situations with ease				
	Willingness to make extra effort to connect in video visits	12 (100)	3.67 (0.492)	4 (3-4)
	Patience with unexpected situations and technological challenges	12 (100)	3.67 (0.492)	4 (3-4)
Respect for patient challenges associated with video visits				
	Respect associated with being in someone’s home digitally	12 (100)	3.83 (0.389)	4 (4-4)
	Respecting patient’s perception of whether video visits can meet their needs and goals	12 (100)	3.67 (0.492)	4 (3-4)
Other				
	Valuing video visits as a viable care delivery model	12 (100)	3.75 (0.452)	4 (3.25-4)
	Receptivity to learning new skills for video visits	12 (100)	3.67 (0.492)	4 (3-4)
	Valuing relational aspects of care with a desire to adapt interpersonal skills for video visits	12 (100)	3.75 (0.452)	4 (3.25-4)

a This describes the percentage of experts who scored the item as either “important” or “very important.”

b Group mean Likert score. Note that each specific item within the survey was rated as 4=very important, 3=important, 2=of less importance, or 1=unimportant.

**Table 5.** Teaching strategies (N=12).

Theme and item		Consensus n (%)^a^	Mean (SD)^b^	Median (IQR)
Experiential learning methods				
	Simulation with standardized patients	11 (92)	3.58 (0.669)	4 (3-4)
	Role-playing with other learners	11 (92)	3.50 (0.674)	4 (3-4)
Real-time feedback				
	Real-time feedback from real and standardized patients	12 (100)	3.75 (0.452)	4 (3.25-4)
	Real-time feedback from educators, colleagues, and mentors	12 (100)	3.75 (0.452)	4 (3.25-4)
	Real-time feedback from artificial intelligence technology (eg, avatars and natural language processing tools)	9 (75)	3.00 (0.953)	3 (2.25-4)
Self-paced learning				
	Asynchronous learning (eg, recorded presentations)	10 (83)	2.92 (0.793)	3 (3-3)
	Videos demonstrating effective and ineffective rapport-building skills	11 (92)	3.50 (0.674)	4 (3-4)
	Module-based learning	9 (75)	3.08 (0.793)	3 (2.25-4)
Small group methods				
	Case studies	11 (92)	3.33 (0.888)	3.5 (3-4)
	Discussion groups	10 (83)	3.00 (0.853)	3 (3-3.75)
	Lecture with breakout practice sessions	10 (83)	3.08 (0.900)	3 (3-4)
Affective learning				
	Opportunities for self-reflection	10 (83)	3.33 (0.985)	4 (3-4)
	Role-playing that includes having the clinician be a patient	10 (83)	3.33 (0.779)	3.5 (3-4)
Other				
	Peer learning (eg, observing, interacting with preceptors, mentors, and role models)	12 (100)	3.58 (0.515)	4 (3-4)
	Integration of telehealth adaptations into standard curricula (ie, when learning about developing in-person rapport, students would also learn about appropriate adaptations for telehealth)	12 (100)	3.92 (0.289)	4 (4-4)

a This describes the percentage of experts who scored the item as either “important” or “very important.”

b Group mean Likert score. Note each specific item within the survey was rated as 4=very important, 3=important, 2=of less importance, or 1=unimportant.

#### Knowledge

Expert participants described the importance of ensuring that students and clinicians had basic knowledge of telehealth. This included the evidence-based uses of VV, types of VV, roles and responsibilities of clinicians, situations not appropriate for VV, common challenges, and required adaptations. Providing information to ensure a functional understanding of videoconferencing technology and how to address common technical problems was also identified as basic telehealth knowledge, as was instruction on specific ways to create a web-based environment supporting rapport. This included creating ground rules for VV and understanding how generational, cultural, educational, geographic, and socioeconomic factors influence a patient’s participation in VV. The importance of ensuring privacy in not only the patient’s but also the clinician’s environment was emphasized as essential to developing rapport and trust. Finally, the experts recommended that basic knowledge of the antecedents (eg, feeling safe and comfortable, privacy, and attentiveness), attributes (eg, shared positive experiences, shared smiles, engagement, and sense of connection), and outcomes (eg, trust, confidence, and collaboration) of rapport were important to teach, along with the unique opportunities and challenges associated with rapport-building in VV.

#### Skills

The data highlighted essential skills for students and clinicians, including creating a safe and comfortable environment, adapting verbal and nonverbal communication techniques for VV, maintaining attentiveness and presence, and using the web-based environment to humanize the care experience. Experts provided detailed recommendations for each category that helped enrich the survey items developed in round 2. For example, they emphasized the importance of establishing rapport at the beginning of a video visit and monitoring it throughout. Managing the time and flow of the VV to avoid rushing patients and preventing abrupt endings were identified as critical skills. Demonstrating cultural humility and navigating moments of interpersonal disconnection were also identified as crucial for maintaining rapport.

Experts highlighted the need to pace verbal communication to reduce interruptions or “talk overs” and to “narrate the visit” by explaining the clinician’s actions that might be unclear or not visible to patients. Using words to replace physical touch to acknowledge emotions and demonstrate care was emphasized. Enhancing active listening techniques, including body language, to reflect empathy and caring was described as particularly important. Examples included placing a hand over the heart to show empathy or leaning into the camera to demonstrate attentiveness.

Participants also stressed the importance of encouraging attentiveness by setting up technology to improve eye contact, sense of presence, and visualization of emotions. Finally, experts noted opportunities within a VV to humanize the experience and build rapport by seeking cues in the patient’s environment to know them as a person and identify common interests. Using unique aspects of a VV, such as unexpected pet encounters or technology issues (eg, sharing a laugh about challenges of technology rather than focusing on frustration), to build interpersonal connection was also described as useful.

#### Attitudes

The importance of cultivating affirming attitudes was mentioned throughout the interview data. This included a willingness to adapt to changing situations with ease, such as making extra efforts to connect in VV and having patience with unexpected situations and technological challenges. Respect associated with entering a patient’s home environment digitally was highlighted, as well as respecting the patient’s perception of whether a VV could meet their needs. Other attitudes that the experts felt needed to be nurtured included valuing VV as a viable care model and adapting relational aspects of care for VV. Being receptive and excited to learn new skills for VV was also emphasized as an important attitude for educators to foster.

#### Teaching Strategies

The importance of integrating telehealth adaptations into the standard educational curriculum was frequently discussed in the interviews, and a variety of teaching strategies were recommended. Methods supporting experiential and affective learning, such as simulation with standardized patients and opportunities for self-reflection, were identified. Self-paced modules, videos demonstrating effective and ineffective rapport-building skills, and small group methods like case studies and discussion groups were identified as useful strategies. The opportunity to receive real-time feedback and learn by observing and interacting with preceptors, mentors, and role models was emphasized.

### Second Round Results

All 75 items that emerged from the qualitative content analysis in round 1 were presented to the experts in a survey format. Participants rated each item’s level of importance from a Likert scale score of 4=very important to 1=not important. The percentage of agreement, the group’s mean Likert score, SD, and IQR for each item follow and are organized by the educational areas: knowledge (Table 2), skills (Table 3), attitudes (Table 4), and teaching strategies (Table 5). All 75 items presented to the experts for rating reached the a priori level of at least 70% consensus in round 2. In total, 71% (53/75) of the items achieved 100% consensus, indicating that all 12 experts felt that these items should be included in a curriculum focused on teaching rapport-building in VV. There were very high levels of consensus among the items related to knowledge and attitude. A total of 20 of 22 (91%) of the items in the knowledge domain had 100% consensus, and all 7 items related to attitudes had 100% consensus. In total, 4 of 15 teaching strategy items reached 100% consensus. Table 6 illustrates the strong levels of consensus achieved in round 2.

**Table 6.** Level of consensus by educational area and teaching strategy.

Educational areas (total items)	Items with 100% consensus, n (%)	Items with 92% consensus, n (%)	Items with 83% consensus, n (%)	Items with 75% consensus, n (%)
Knowledge (n=22)	20 (91)	1 (5)	0 (0)	1 (5)
Skills (n=31)	22 (71)	4 (13)	3 (10)	2 (7)
Attitudes (n=7)	7 (100)	0 (0)	0 (0)	0 (0)
Teaching strategies (n=15)	4 (27)	4 (27)	5 (33)	2 (13)

The group’s mean Likert scores ranged from 2.83 to 4.00 (SD 0-0.985) for each item. The high mean of most of the items also reflects strong agreement on the items evaluated in round 2. Although there were no disputed items, 5 items only reached a 75% consensus level (Table 6). The high levels of consensus resulted in most items having relatively low SD and IQR ratings.

Three items (taxonomy, asynchronous learning, and discussion groups) in particular had lower means, with higher SDs. The median of these items was 3 (IQR 2.25-3), 3 (IQR 3-3), and 3 (IQR 3-3.75), respectively. This suggests that a core group of the experts closely agreed, but a few participants felt differently about these 3 items. The median for real-time feedback with AI was 3 (IQR 2.25-4), and the median for modular learning was 3 (IQR 2.25-4). Both also had high SDs (Table 5). This suggests that these items were more complex or controversial with a broader range of opinions, even though they still met the consensus threshold.

Open-ended responses from several of the experts in this round suggested the need for 2 additional items. This included the importance of incorporating information on ways a clinician could adapt teaching methods in VV and providing opportunities for practicing VV that involved an interprofessional team.

### Third Round Results

Since all 75 items in round 2 reached the established consensus threshold, round 3 focused on gathering feedback from the expert panel on the 2 new items developed from the open-ended survey responses in round 2. Consistent with round 2, these items were presented in a Qualtrics survey, with experts asked to rate their importance on a 4-point Likert scale. Both items met the consensus threshold. The first item, “importance of adapting teaching methods to patient learning preferences and experience with videoconferencing,” had 100% (12/12) consensus, a mean Likert score of 3.58 (SD 0.52), and a median of 4 (IQR 3-4). The second item evaluated in round 3, “opportunities to practice VV involving an interprofessional team,” achieved 92% (11/12) consensus, a mean Likert score of 3.17 (SD 0.58), and a median of 3 (IQR 3-3.75). Open-ended feedback in round 3 suggested distinguishing educational content on patient learning preferences from content on the patient experience with VV, as these are distinct topics. Experts also noted that some patient learning preferences may not be viable options in VV.

### Fourth Round Results

The focus of round 4 was to share the study’s overall results and provide the experts an opportunity to indicate if these accurately reflected their experiences and perspectives. The participants’ responses affirmed the results, and as one expert shared, “Building rapport through video-mediated communication can be challenging, and the items highlighted tangible and effective steps to mitigate this.” Some of the experts’ responses helped explain areas where there was less than 100% consensus. One expert indicated that in the populations they worked with, patients may not be comfortable with clinicians stating, “I wish I could offer you a hug” or having clinicians humanize a VV by looking for things in their environment. These comments reflected the importance of cultural humility, understanding the context of the VV, and individual patient preferences and perspectives. One expert expressed surprise that having students role-play being a patient did not receive a higher level of consensus sharing, “Empathy is a muscle. If you don’t consistently remind yourself what it feels like to be the patient, you are more likely to forget and deliver more callous, depersonalized care.”

All the participants indicated that they felt the results could inform interprofessional practice and teaching. As one expert stated,

These results can be a great jumping-off point for developing more focused training and education around telehealth communication. Since there seems to be a general awareness of the importance of rapport, but a lack of clarity on the specific skills needed, there’s an opportunity to design tools, workshops, or even curricula that help providers build those skills more intentionally.

Another shared, “This could really push institutions to start thinking beyond the technology and more about the patient experience.”

## Discussion

### Principal Findings

**Figure 1.**
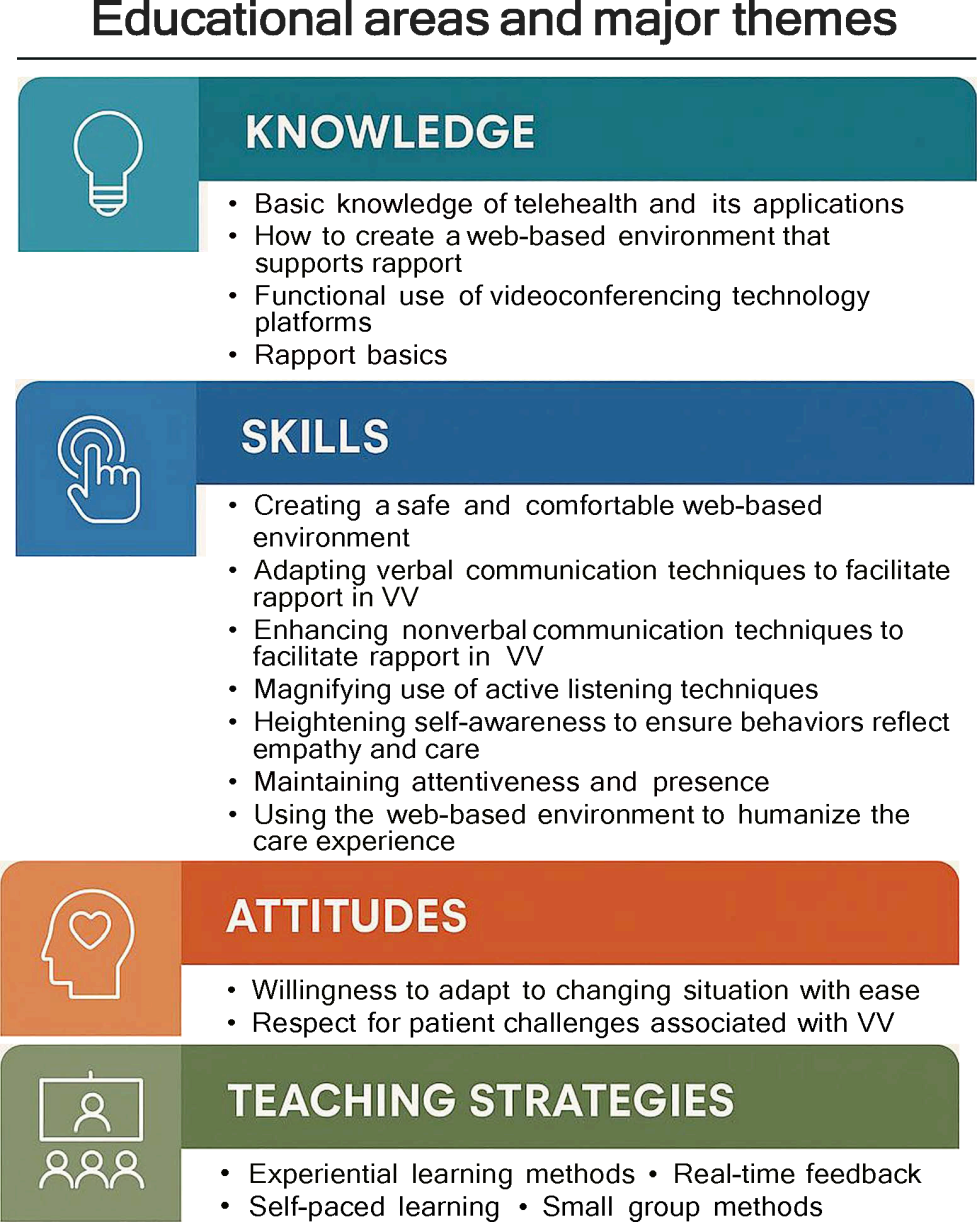
Summary of major themes. VV: videoconferencing visit.

Teaching and learning how to establish and maintain rapport in VV were identified as important educational gaps. The aim of this study was to build consensus around the knowledge, skills, and attitudes required for health professions students and clinicians to build rapport with patients in VV as well as to identify what teaching strategies best support these educational objectives. This e-Delphi approach was an effective and efficient method for consolidating the expertise of 12 interprofessional telehealth clinicians and educational experts (Figure 1). The high retention rate throughout the study may indicate the importance and relevance of this specific topic to telehealth experts.

The experts quickly agreed on the importance of 19 themes and 77 items to guide curriculum development. The depth and breadth of these themes and items demonstrate the importance of enhancing relationship skills beyond those described in etiquette guidelines or competencies. Although the number of themes and items could be perceived as excessive or impractical to incorporate into telehealth education, evidence that clinicians lack confidence in their relationship-based skills demonstrates the need for a more robust curriculum. The study results provide a comprehensive and useful reference for building a curriculum based on developing knowledge, skills, and attitudes. Like other complex competencies, these may need to be integrated throughout a health profession curriculum or offered in self-paced modules for professional development.

The number of themes and items related to adapting relationship-based care and communication skills for VV demonstrates the importance of this element in the curriculum and aligns with existing research [13,14,20,29]. The expert panel also emphasized the importance of learning to create interpersonal connections at the beginning of a VV and monitor the level of rapport throughout the visit. Additionally, not rushing or ending the VV abruptly and taking time to clearly understand the patient’s wants and needs with mutually agreeable care goals were highlighted as important to building rapport. These suggestions align with telehealth best practices reported in oncology and palliative care [29,31,44], as well as practices identified as important in ambulatory care [10,13,45,46].

Similarly, fostering a sense of presence and attentiveness during the VV was a key focus. Many of these items have been reported in other research as important, including how to set up technology to enhance telepresence and body language communication (eg, eye contact, facial expressions, and body posture) and paying close attention to visual and auditory signals that might reflect patient emotion [20,26,47]. Best practices related to camera positioning and eye gaze originally recommended by the American Telemedicine Association [48] have been shown to influence interpersonal comfort and self-disclosure in web-based interactions and may facilitate trust and collaboration [49].

The curricular themes and items also addressed navigating barriers that impact building rapport in VV. Rettinger and Kuhn [50] described internet connectivity issues and a lack of skills in navigating or problem-solving technological challenges in VV as the barriers most frequently described in the 56 studies included in their scoping review. What was highlighted by the experts in this e-Delphi study is the importance of managing these challenges to maximize clinician-patient rapport. In addition to problem-solving skills, the overall attitude of the clinician in managing these challenges was highlighted. The experts felt that patience and light-heartedness were essential attitudes, even suggesting that technological challenges could present opportunities to build rapport around a shared experience. The high level of importance expressed by the expert panel for teaching rapport basics and managing VV challenges affirms the need for clinicians to be proficient and comfortable in both interpersonal and technological skills.

A recent scoping review on rapport in oncology ambulatory care found that more studies focused on nurses’ attitudes than knowledge and skills [51]. Attitudes were also identified as critical by the experts in this study, who indicated that without positive attitudes, motivating adult learners to acquire new knowledge and skills becomes challenging. They articulated the need to cultivate positive attitudes toward telehealth and a willingness to adapt interpersonal skills accordingly. This was also identified as an important barrier in the scoping review of Rettinger and Kuhn [50].

Affective competency is reflected in students’ ability to conceptualize and internalize subject matter related to attitudes, emotions, or biases in ways that influence their values, decisions, and behaviors [52,53]. Teaching in this domain of learning requires that educators place a greater emphasis on their students becoming caring, emotionally and culturally intelligent, and ethical professionals rather than on their acquisition of factual knowledge [20,53,54]. Achieving this goal necessitates creating a safe learning environment and using teaching strategies that enhance student self-awareness and encourage exploration of diverse perspectives and needs [53,55]. This highlights the importance of building a curriculum and using teaching strategies that address the affective domain of learning and aligns with the importance the experts placed on attitudes and reflective teaching strategies.

Research suggests that opportunities to practice conducting VV with standardized patients and in simulation laboratories have promoted positive learning outcomes [56]. This aligns with the results of this study, where real-time feedback and experiential learning methods reached higher levels of consensus. While module-based learning is evidence-based and necessary, given that this is how many health educational and professional programs are currently delivered [57], the experts in this study emphasized the importance of a learning environment that enabled hands-on practice.

Our results are relevant to various health professions that engage in telehealth. The Interprofessional Education Collaborative (IPEC) Core Competencies for Interprofessional Collaborative Practice [58] provide a framework for interprofessional collaborative practice centered upon 4 competency domains: communication, teams and teamwork, roles and responsibilities, and values and ethics. Each of the 4 competency domains is well-aligned with our panel’s insights into effective education for telehealth rapport-building. Specifically, the IPEC competencies in communication and teams and teamwork resonate with our expert panel’s emphasis on clear, culturally humble, and empathic communication adapted to the web-based setting. IPEC competency statements regarding communication that is responsive, respectful, and compassionate, as well as practicing active listening, directly relate to skills our expert panel prioritized, such as narrating visits, managing disruptions, and adapting both verbal and nonverbal cues to build rapport. The roles and responsibilities IPEC competency domain align with the panel’s suggestion to teach interprofessional learners how to understand one another’s scopes of practice and leverage each team member’s unique contributions to enhance web-based care experiences. Finally, the values emphasized throughout the IPEC values and ethics domain, which include respect, cultural humility, and patient-centeredness, mirror the attitudinal attributes our experts identified as critical to building trust and interpersonal connection in video visits. The high level of consensus across the Delphi rounds and the incorporation of strategies like team-based practice opportunities, patient privacy, and rapport measurement underscore the practical application of IPEC competencies when designing meaningful telehealth education for various health professions.

### Implications and Future Research

The research questions and analysis used a competency-based educational framework often applied by educators when developing competency-based education [59]. Organizing the findings by cognitive or mental skills (knowledge), manual psychomotor skills (skills), and affective skills (attitudes) was done intentionally to make it relevant and easy for health profession educators to incorporate the results into their existing competency-based telehealth programs. When paired with existing checklists [25,26,46] and professional competency guidelines [3,4,58], these results can support the creation of stand-alone programs on relationship-based care in telehealth or module-based programs that can be integrated into broader telehealth curricula.

Future research is needed to test the feasibility, acceptability, and effectiveness of building curricula based on these competencies. Pilot testing of the proposed curriculum model would be an important next step in evaluating learning outcomes. This could be followed by a controlled study to examine whether these outcomes translate into measurable improvements in perceived rapport scores. In addition, gaining a better understanding of the patient perspective on rapport-building strategies is an important area for further study, along with exploring how these strategies may vary based on the VV context, purpose, and the type of professional conducting the VV.

### Strengths and Limitations

The strengths of this study include the collection of data from interprofessional health care clinicians and educators. The use of the ACCORD [43] and multiple strategies to enhance the trustworthiness bolstered methodological rigor. The analysis incorporated a broad range of participants’ responses to ensure credibility. Offering participants opportunities in rounds 2‐4 to review the findings and provide feedback or suggestions also enhanced the study’s dependability. The context of the participants was also thoroughly described to allow readers to determine how the findings can be applied in other programs and settings. Although the goal of a Delphi study is to build consensus, there is a risk of forcing conformity [32]. By interviewing participants individually in the first round, this study provided each expert an opportunity to fully share their views and ensured the participants remained anonymous to one another. The rapid and high levels of consensus for most of the items suggest that the analysis of the interview data and subsequent development of survey items were dependable, largely representing the experts’ opinions.

This study has several limitations. Given the limited existing research on building rapport in VV, the reliance on expert interviews in round 1 constrained the participant pool. Although purposive criteria guided recruitment, the panel did not encompass all professions engaged in the use and instruction of VV. As such, the panel’s composition may not fully reflect the broader population of telehealth clinicians and educators, particularly those in early-career stages. In addition, most participants were based in the United States, which may limit the applicability of findings in international or cross-cultural contexts. This may stem from social and cultural differences in communication norms. In addition, variations in health care systems, technological proficiency, device access, and internet availability across countries may further limit the generalizability of the findings. Finally, although there are no strict guidelines for Delphi panel size, the relatively small sample may not have captured the full range of perspectives. These factors may have increased the risk of selection bias and could affect the validity and generalizability of the study findings [32].

### Conclusions

It is widely recognized that a strong rapport between patients and clinicians is essential in VV. Yet, despite this understanding, evidence-based practical guidance on adapting interpersonal skills for web-based care remains limited. This study offers a tangible first step toward addressing this gap. Its results provide detailed guidance on the knowledge, skills, and attitudes necessary for health professions students and clinicians to build rapport in VV settings. In addition to outlining these competencies, the study presents a clear structure, actionable content, and recommended teaching strategies to support curriculum development in health professions education. While web-based care brings numerous benefits, failing to deliberately adapt relationship-based skills for VV risks reducing patient interactions to impersonal, task-focused exchanges. To fully realize the potential of telehealth, it is critical to invest in evidence-based approaches that preserve and promote meaningful interpersonal connections in the web-based environment—for the benefit of both patients and clinicians.

## Abbreviations

ACCORD: Accurate Consensus Reporting Document
AI: artificial intelligence
IPEC: Interprofessional Education Collaborative
VV: videoconferencing visit
